# Supplementary material for: Enhancement of VGG16 model with multi-view and spatial dropout for classification of mosquito vectors
Source: PLoS One. 2023 Jul 24;18(7):e0284330. doi: 10.1371/journal.pone.0284330 (PMC10365266; doi:10.1371/journal.pone.0284330)
Supplement: Attachment — Submitted filename: RebuttalLetter.docx [file pone.0284330.s003.docx]

February 26, 2023

*Re: PLOS ONE Decision: Revision required [PONE-D-22-32464] - [EMID:558653f01c3748f5]*

Dear reviewer:

Thank you very much for your effort and time to read and comment on our manuscript entitled “Enhancement of VGG16 model with multi-view and spatial dropout for classification of mosquito vectors.” We really appreciate your kind comments.

We would like to respond to your comments (in gray italics) as follows:

1. Would you please mention the resolution of the high-quality photographs mentioned in the subchapter "Image datasets"?
   Thank you so much for bringing this to our attention. We added a sentence: The 64MP smartphone was set to produce photos at its highest resolution of 9,248 x 6,936. Hopefully, it will provide additional information to the audience.
2. What is the resolution of the photographs captured by the mid-range and low-end smartphones mentioned in the subchapter "Image datasets"?
   Again, thank you for pointing this out. So we added a sentence: "The 64MP mid-range and the 13MP low-end phones produced images with resolutions of 9,248 x 6,936 and 4,128 x 3,096, respectively." To remind the audience that the quality of the image does not depend solely on the image resolution, we added further a sentence: "Note that the quality of the lens and image sensors on the phones' cameras has a greater impact on the resulting photos than the resolutions of the cameras." Despite having the same image resolution, the Vivo V21 and Samsung A52s received DXOMark (still camera) scores of [105](https://www.dxomark.com/vivo-v21-5g-camera-review-good-stills-in-its-segment/) and [88](https://www.dxomark.com/smartphones/Samsung/Galaxy-A52s-5G), respectively. This shows that the cameras on the Vivo V21 are generally better than those on the Samsung A52s. Note that DXOMark.com did not give the Vivo Y21 a camera test.

We hope that our revision will address your concerns and that it is good enough to be published in the prestigious PLOS ONE journal.

Sincerely yours,

Corresponding author on behalf of all authors
